# α-Gal on the protein surface affects uptake and degradation in immature monocyte derived dendritic cells

**DOI:** 10.1038/s41598-018-30887-8

**Published:** 2018-08-23

**Authors:** M. Krstić Ristivojević, J. Grundström, T. A. T. Tran, D. Apostolovic, V. Radoi, M. Starkhammar, V. Vukojević, T. Ćirković Veličković, C. Hamsten, M. van Hage

**Affiliations:** 1 Department of Medicine Solna, Immunology and Allergy Unit, Karolinska Institutet, and University Hospital, Stockholm, Sweden; 2 Center of Excellence in Molecular Food Sciences, Faculty of Chemistry, University of Belgrade, Belgrade, Serbia; 3 Department of Clinical Neuroscience, Center for Molecular Medicine (CMM), Karolinska Institutet, Stockholm, Sweden; 4 Department of Internal Medicine, Södersjukhuset, Stockholm, Sweden; 5 Ghent University Global Campus, Yeonsu-gu, Incheon, South Korea

## Abstract

Red meat allergy is characterized by an IgE response against the carbohydrate galactose-α-1,3-galactose (α-Gal), which is abundantly expressed on glycoproteins from non-primate mammals. The mechanisms of how α-Gal is processed and presented to the immune system to initiate an allergic reaction are still unknown. The aim of this study was to reveal whether the presence of α-Gal epitopes on the protein surface influence antigen uptake and processing in immature monocyte-derived dendritic cells (iMDDCs). Immature MDDCs were prepared from healthy blood donors and red meat allergic patients. We found an increased internalization of α-Gal carrying proteins over time in iMDDCs by flow cytometric analysis, which was independent of the donor allergic status. The uptake of α-Gal carrying proteins was significantly higher than the uptake of non-α-Gal carrying proteins. Confocal microscopy revealed α-Gal carrying proteins scattered around the cytoplasm in most iMDDCs while detection of proteins not carrying α-Gal was negligible. Fluorescent detection of protein on SDS-PAGE showed that degradation of α-Gal carrying proteins was slower than degradation of non-α-Gal carrying proteins. Thus, the presence of α-Gal on the protein surface affects both uptake and degradation of the protein, and the results add new knowledge of α-Gal as a clinically relevant food allergen.

## Introduction

Allergy to red meat presents as a delayed early phase allergic reaction, usually 3–6 hours after red meat consumption^1–3^. The reaction is due to IgE antibodies toward the oligosaccharide galactose-α-1,3-galactose (α-Gal)^1–4^, which is expressed on non-primate mammalian proteins. In humans and higher primates, the gene encoding α-1, 3-galactosyltransferase is non-functional and thus α-Gal is not expressed^5^. Induction of red meat allergy has been linked to tick bites in many parts of the world and α-Gal has shown to be present in ticks^4,6–8^, but the mechanism of sensitization is still unknown. The unusually long delay of symptoms is also not fully understood, but is presumably linked to delayed appearance of the antigen in the bloodstream and/or processing by dendritic cells (DCs) and T-cells.

Dendritic cells are professional antigen presenting cells and immature DCs are sampling the environment to capture antigens and initiate an adaptive immune response by activating and priming naive CD4^+^ T-cells^9^. The subsequent outcome of this interaction can be tolerance promotion or induction of active immunity^10^. Internalization and degradation of antigens by DCs are important steps in determining whether an antigen will be treated as dangerous or innocuous^11^. Antigens can be actively internalized in several different ways: receptor-mediated endocytosis, macropinocytosis and phagocytosis^12^. After uptake, the vesicle formed fuses with endosomal compartments where the antigens will be processed into peptides that can be further presented on the cell surface.

In this study we investigated whether the presence of allergenic α-Gal epitopes on the protein surface affects internalization by immature monocyte derived DCs (iMDDC). Furthermore, we analyzed the uptake in iMDDCs from both healthy blood donors and red meat allergic patients, as well as the effect of protein size and type of carbohydrate on protein uptake.

## Results

### α-Gal glycosylation influences protein uptake

**Figure 1:**
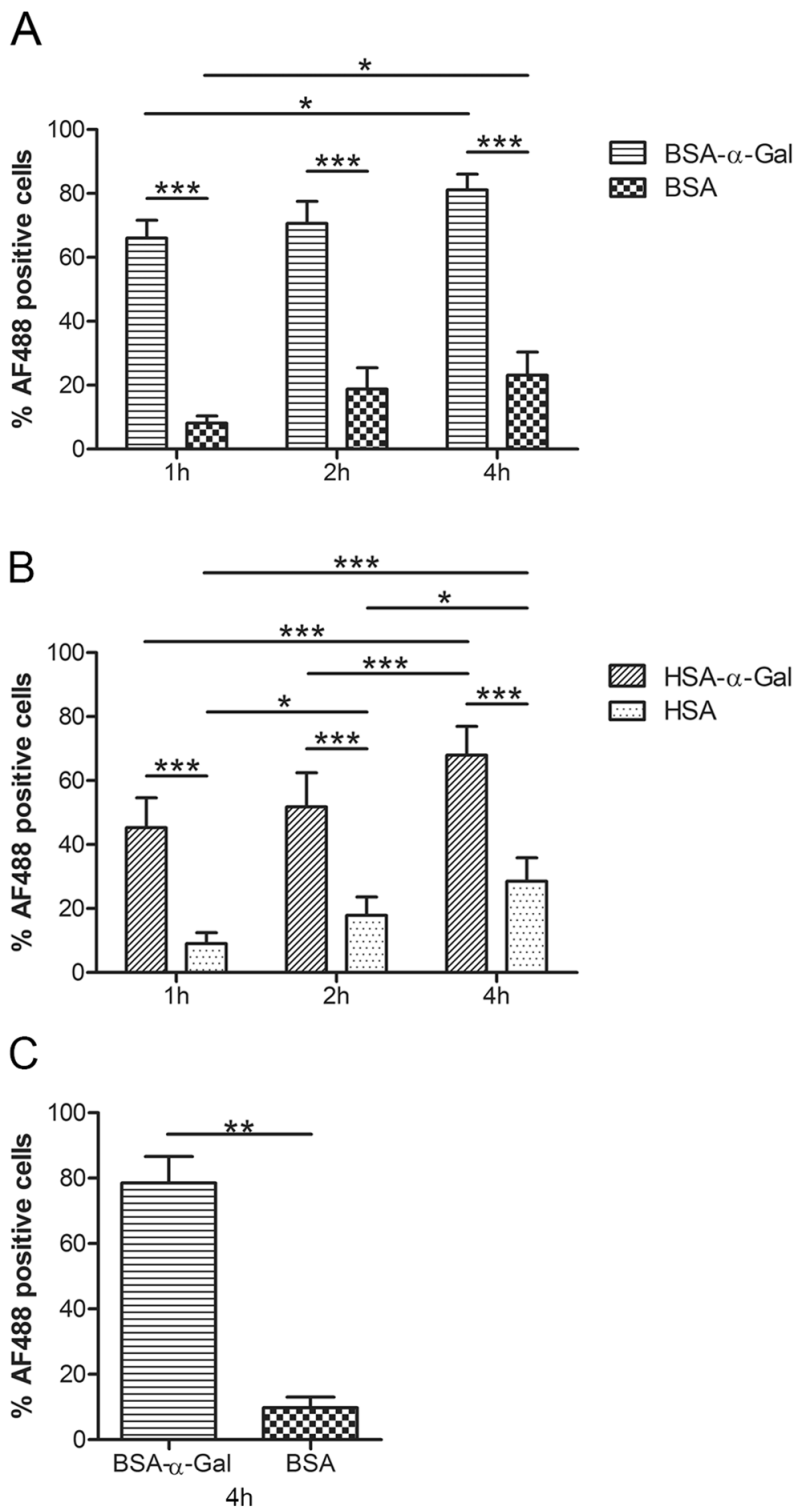
Time dependent uptake of (**A**) BSA-α-Gal and BSA, n = 10, and (**B**) HSA-α-Gal and HSA, n = 8, by iMDDCs generated from healthy donors. *p < 0.05, and ***p < 0.001 analyzed by two-way ANOVA with Bonferroni’s post hoc test. (**C**) Uptake of BSA-α-Gal and BSA by iMDDCs generated from red meat allergic patients after 4 h of incubation, n = 4. **p < 0.001 analyzed by paired t-test.

Uptake of fluorescently labeled α-Gal and non-α-Gal carrying proteins by iMDDCs differentiated from healthy individuals was analyzed after 1, 2 and 4 h. Accumulation of bovine serum albumin carrying α-Gal (BSA-α-Gal) was observed over time (p = 0.0107, from 1 h to 4 h) and internalization of BSA-α-Gal was significantly higher compared to internalization of BSA at all three time points (p < 0.0001, Fig. 1A). Similarly, uptake of human serum albumin carrying α-Gal (HSA-α-Gal) was significantly higher compared to the uptake of HSA (p < 0.0001 at all time points), and internalization of both proteins increased over time (HSA-α-Gal: p < 0.0001 from 1 to 4 h and p = 0.0002 from 2 to 4 h, HSA: p = 0.0441 from 1 to 2 h, < 0.0001 from 1 to 4 h and 0.0127 from 2 to 4 h, Fig. 1B). Uptake of fluorescently labeled BSA-α-Gal and BSA by iMDDCs from four red meat allergic patients was analyzed after 4 h of incubation. Comparably to healthy individuals, BSA-α-Gal was internalized to a significantly higher degree than BSA (p = 0.0013, Fig. 1C).

**Figure 2:**
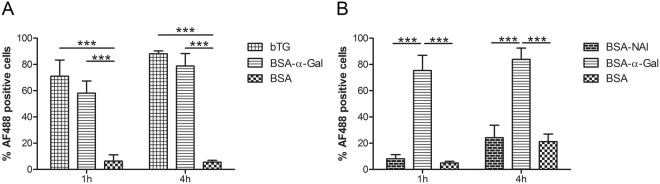
The impact of protein size and conjugation on protein uptake. The uptake of bTG compared to the uptake of BSA-α-Gal and BSA. (**B**) The uptake of BSA-NAl compared to the uptake of BSA-α-Gal and BSA. iMDDCs were generated from healthy blood donors, n = 4. ***p < 0.001 analyzed by two-way ANOVA with Bonferroni’s post hoc test.

To investigate whether protein size and the type of carbohydrate modification carried by the protein affect iMDDC uptake, cells from healthy donors were also stimulated with bovine thyroglobulin (bTG), with a molecular weight 10 times higher than BSA-α-Gal, and BSA-N-Acetyllactosamine (NAl), a carbohydrate modification with similar size as α-Gal. (Fig. 2). Uptake of bTG was comparable to uptake of BSA-α-Gal, showing significantly higher uptake compared to BSA at 1 and 4 h (p < 0.0001, Fig. 2A). However, the uptake of BSA-NAl was comparable to BSA and significantly lower compared to the uptake of BSA-α-Gal after 1 and 4 h of incubation (p < 0.0001, Fig. 2B).

### Inhibition of internalization routes

**Figure 3:**
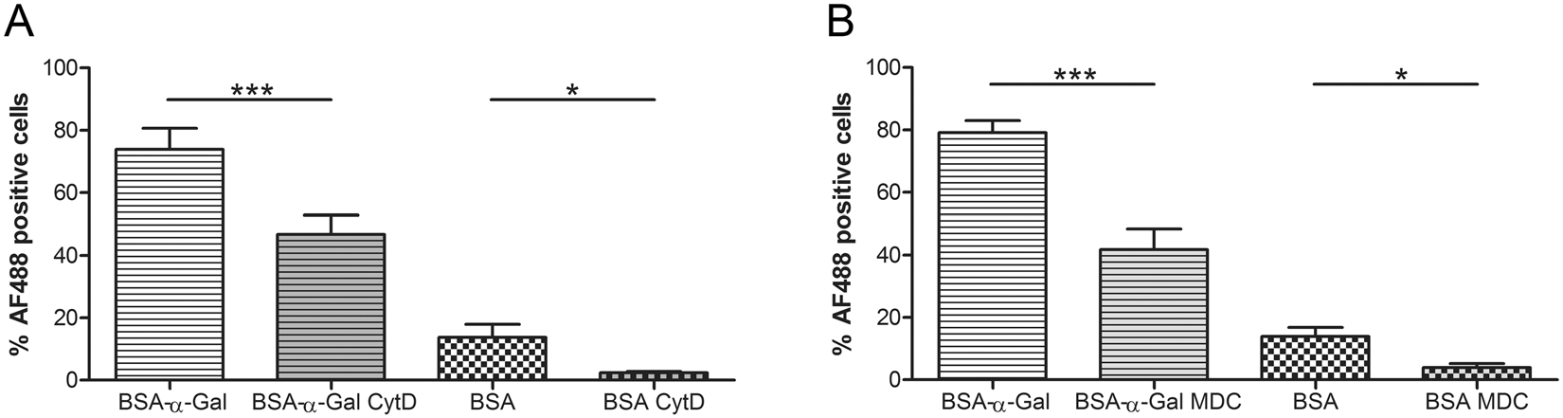
Inhibition of internalization of BSA-α-Gal and BSA in healthy iMDDCs after 1 h of incubation by (**A**) Cytochalasin D, n = 6 (BSA-α-Gal) and n = 5 (BSA), and (**B**) Monodansylcadaverine, n = 7. *p < 0.05, ***p < 0.001 analyzed by paired t-test.

**Figure 4:**
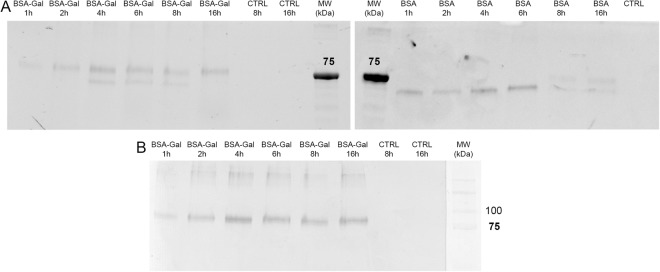
(**A**) Fluorescent detection of BSA-α-Gal and BSA in iMDDCs lysates resolved on an SDS-polyacrylamide gel. (**B**) Western blot detection of the α-Gal epitope in lysates from iMDDCs. iMDDCs were generated from healthy blood donors and unstimulated cells were used as control. Full length gels and blot are presented in Supplementary Fig. S2.

The uptake of α-Gal carrying proteins was confirmed to be an active process, since internalization was significantly reduced by incubating the cells at 4 °C for 4 h (BSA-α-Gal, p = 0.0017, and BSA, p = 0.0165, Supplementary Fig. S1A). To analyze by which route α-Gal carrying proteins are internalized, the inhibitory agents cytochalasin D (CytD), which inhibits macropinocytosis, and monodansylcadaverine (MDC), which inhibits receptor-mediated endocytosis, were added to the iMDDC cultures 30 min before addition of BSA-α-Gal or BSA. Incubation of iMDDCs with both CytD and MDC inhibited the internalization of BSA-α-Gal and BSA at 1 h (CytD: BSA-α-Gal, p = 0.0001, and BSA, p = 0.0397, and MDC: BSA-α-Gal, p = 0.0001, and BSA, p = 0.0385, Fig. 3) and 4 h (CytD: BSA-α-Gal, p = 0.0156, and BSA, p = 0.0327, and MDC: BSA-α-Gal, p < 0.0001, and BSA, p = 0.0018, Supplementary Fig. S1B and C).

### The α-Gal epitope can be detected in iMDDC lysates

Immature MDDC lysates from cells harvested at different time points were analyzed by SDS PAGE and Western blot for the presence of the α-Gal epitope. Detection of fluorescently labeled proteins showed a band above 75 kDa that was present for up to 16 h of uptake of BSA-α-Gal (Fig. 4A). After 4, 6 and 8 h of BSA-α-Gal incubation, a protein band lower than the intact protein was detected, which probably represents degraded products of the protein. For BSA, a strong protein band was detected for up to 6 h of uptake (Fig. 4A). Western blot analysis confirmed that the α-Gal epitope was present in cell lysates from iMDDCs incubated with BSA-α-Gal for up to 16 h (Fig. 4B). For size comparison, the pure proteins (BSA and BSA-α-Gal) were separated onto 12% polyacrylamide gel via SDS-PAGE (Supplementary Fig. S3).

### α-Gal containing proteins are scattered in the cytoplasm of iMDDCs

**Figure 5:**
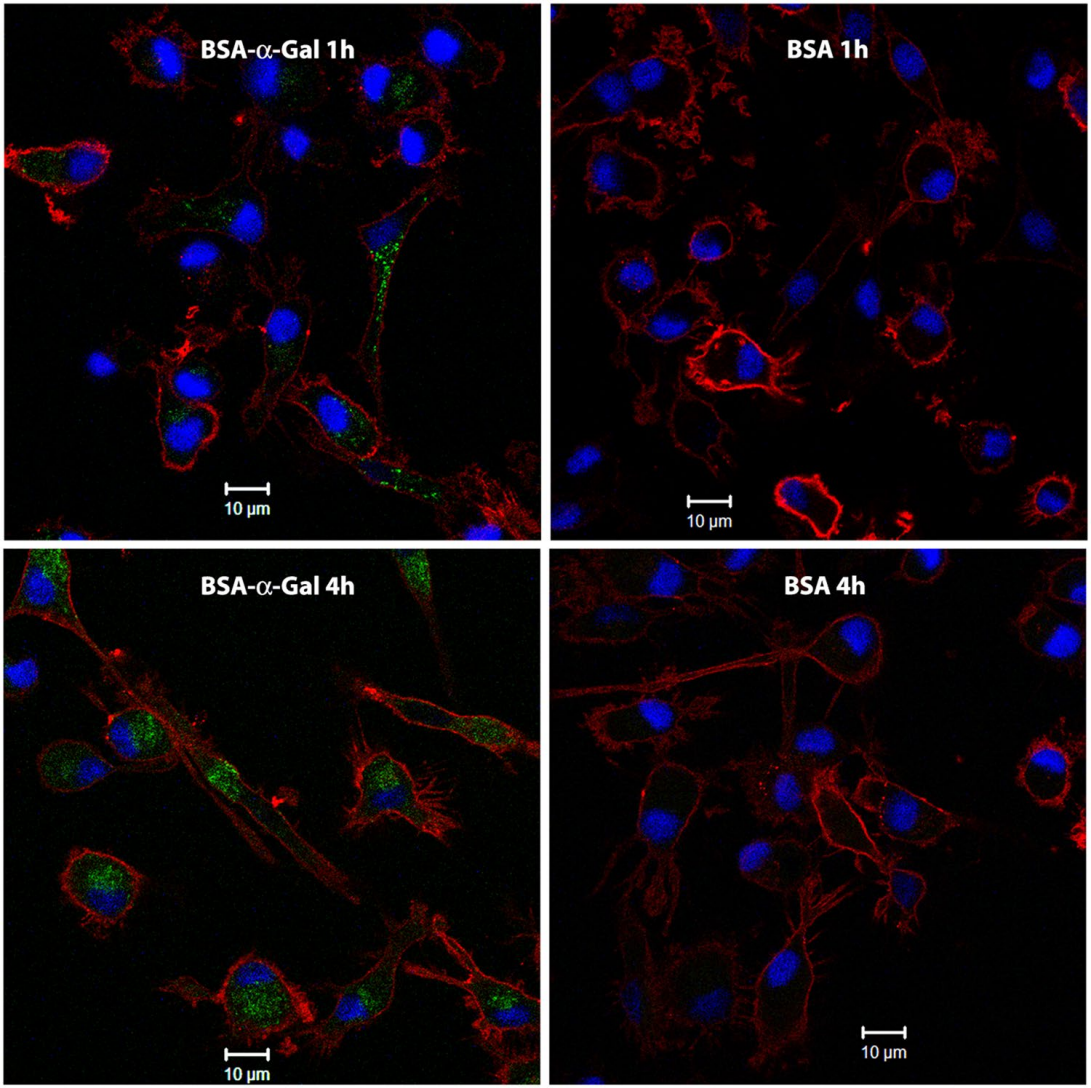
Uptake of BSA-α-Gal (left) and BSA (right) after 1 h (top) and 4 h (bottom) of iMDDC incubation at 37 °C analyzed by confocal laser scanning microscopy. Green = BSA-α-Gal or BSA, red = HLA-DR and blue = DAPI stained nuclei.

Confocal laser scanning microscopy imaging revealed that after 1 h of incubation, BSA-α-Gal was present at detectable levels in the cytoplasm in about 50% of the iMDDCs. After 4 h of incubation it was observed in all cells, with a prevalent localization in the perinuclear region (Fig. 5, green). In contrast, BSA uptake by iMDDCs was not detected, either after 1 or 4 h of incubation, evidenced by the lack of green signal (Fig. 5). Observations made by imaging confirmed the flow cytometric analysis. There was no detectable uptake of BSA-NAl after 1 and 4 h of incubation, whereas after 1 and 4 h of incubation with the α-Gal containing protein bTG, the fluorescently labeled protein was scattered in the cytosol of almost all iMDDCs (Supplementary Fig. S4).

## Discussion

α-Gal rich foods elicit delayed allergic symptoms in red meat-allergic patients, which could be due to uptake and processing by dendritic cells. In the present study we show efficient uptake of the α-Gal model antigens BSA-α-Gal and HSA-α-Gal by *in vitro* cultured human iMDDCs. Similar results were obtained for iMDDCs generated from healthy individuals and red meat allergic patients, indicating that altered uptake of α-Gal in dendritic cells *per se* is not a factor contributing to red meat allergy. However, DCs are likely involved in the sensitization phase of red meat allergy where α-Gal containing proteins may be taken up in the close vicinity of a tick bite. In atopic individuals the DCs may then initiate allergic sensitization through skewing of CD4+ T cells towards a Th2 response. A recent report showed that the context of allergen uptake is important for maturation of iMDDCs and that the maturation effect differed between healthy and atopic donors^13^.

Interestingly, the uptake of BSA and HSA alone was not as efficient as for their α-Gal carrying counterparts. Thus, the presence of α-Gal on the surface seems to enhance internalization of the protein, suggesting an α-Gal dependent uptake mechanism. The results are in line with previous studies on glycosylated protein uptake, where a 100-fold higher uptake of mannosylated BSA compared with non-mannosylated BSA was demonstrated^14^. Furthermore, advanced glycation end products of ovalbumin were taken up more efficiently by iMDDCs compared with ovalbumin^15^.

We also investigated if the size and conjugation of the proteins have impact on the uptake. Immature MDDCs from healthy individuals showed no difference in the uptake of BSA-α-Gal and bTG even though the molecular weight of bTG is approximately 10 times higher than the molecular weight of BSA-α-Gal. Interestingly, the uptake of BSA-NAl was several times lower compared to BSA-α-Gal, even though the α-Gal and NAl carbohydrates are of similar size. Previous studies have shown that MDDCs internalize BSA with different glycosylation patterns to very varying extent^16^, and for other glycosylated allergens the mannose receptor plays a major role in glycoallergen recognition and uptake^17^. Our results strongly suggest that the specific glycosylation of protein antigens predominantly influence the uptake, while the size is not a decisive factor. This is in line with previous studies which have shown that human DCs, in addition to protein antigens, are able to internalize large size particles^18^ while the key factor that influence protein uptake is the surface architecture^14^.

We next elucidated whether α-Gal could be detected in the cytoplasm of iMDDCs. Fluorescence detection confirmed the presence of BSA-α-Gal in iMDDC cytoplasm. Moreover, SDS PAGE analysis showed that BSA-α-Gal was detected after up to 16 h of incubation, while BSA was only detected up to 6 h. Western blot with monoclonal anti-α-Gal antibody confirmed that the BSA-α-Gal still carries the epitope. Our results suggest that the degradation pathway of α-Gal carrying proteins is slower than for non-carrying proteins and that they are stored intact inside of cells. Most likely the α-Gal glycosylation protects the protein from degradation and as such may increase the processing time and exposure on the surface of the cell. Other studies have indeed shown that reduced degradation increases antigen presentation on MHCII^19^ and that the allergenic Bet v 1a was degraded slower than its hypoallergenic isoform Bet v 1d^20^, suggesting that processing time affects allergenicity. This is also in line with the delayed appearance of symptoms in red meat allergic patients.

The route of internalization was analyzed by adding CytD and MDC to the iMDDC cultures which resulted in a partial inhibition of the internalization of BSA, regardless of the presence of α-Gal. The inhibition was stronger after 1 h of incubation than after 4 h, which probably reflects a temporary effect by both inhibitory agents. MDC blocks receptor-mediated endocytosis whereas CytD affects invagination of the cell membrane by blocking actin polymerization, which inhibits macropinocytosis and phagocytosis^21^. However, blocking of actin polymerization may also inhibit receptor-mediated endocytosis^21^. Fluorescently labeled BSA can be used for measuring uptake via macropinocytosis, but as with chemical inhibition of antigen-uptake, it is difficult to unambiguously distinguish between macropinocytosis and receptor-mediated endocytosis as the uptake mechanism in DCs^22^. Our results thus indicate that both receptor-mediated endocytosis and macropinocytosis are involved in the uptake of α-Gal containing proteins.

The confocal analysis revealed accumulated BSA-α-Gal and bTG after 1 and 4 h of incubation, whereas no internalization of BSA and BSA-NAl could be detected at any time point. Thus, for antigen uptake the conjugated sugar seems to be more important than the protein size, in line with the flow cytometric analysis. After 1 h the proteins were found in clusters scattered around the cytoplasm while after 4 h, the proteins were found closer to the nucleus, suggesting that BSA-α-Gal is taken up and processed in endosomes.

In conclusion, α-Gal containing proteins are actively and promptly internalized by iMDDCs in a time-dependent manner, and the mechanism of internalization seems to be α-Gal dependent. Thus, α-Gal on the protein surface affects uptake and processing of the protein, and the results add new knowledge of α-Gal as a clinically relevant food allergen.

## Methods

### Reagents

BSA, HSA, bTG, MDC and CytD were obtained from Sigma-Aldrich (Saint Louis, MO, USA). BSA-α-Gal, HSA-α-Gal, and BSA-NAl were obtained from Dextra Laboratories (Reading, UK).

### Fluorescent labelling of proteins

BSA, BSA-α-Gal, BSA-NAl, HSA, HSA-α-Gal and bTG were fluorescently labeled with Alexa Fluor 488 (AF 488) using the protein labelling kit (Thermo Fisher, Rockford, IL, US) according to the manufacturer’s instructions. Protein concentration was determined using the bicinchoninic acid protein assay kit (Life technologies, Carlsbad, CA, USA) according to the manufacturer’s instructions. The endotoxin levels were determined by the limulus amebocyte lysate assay (Charles River Endosafe, Charleston, SC, USA) to less than 1 ng LPS/mg of protein in all protein preparations. The labeled proteins were stored at −20 °C and protected from light until further use.

### Donors and patients

Buffy coats from 18 healthy blood donors and four patients diagnosed with red meat allergy by a physician experienced in allergic diseases were included in the study.

### Ethics Statement

The study was approved by the local ethics committee (2014/847-32 and 2016/1447-32) and all experiments were in accordance with relevant guidelines and regulations. Sample collection was done after written informed consent from the study participants.

### Cell isolation and culturing

Peripheral blood mononuclear cells were isolated by density gradient centrifugation using Ficoll–Paque (GE Healthcare, Uppsala, Sweden). CD14^+^ monocytes were isolated by magnetic bead separation (Miltenyi Biotec, Bergisch Gladbach, Germany) in accordance with the manufacturer’s instructions. Immature MDDCs were generated as previously described^23^. For uptake analysis, iMDDCs were seeded in multiwell flat-bottom tissue culture plates and incubated with 10 µg/10^6^ cells of fluorescently labeled proteins for 1, 2 and 4 h. Internalization was inhibited by incubating the iMDDCs at 4 °C, or by adding MDC (200 µM) or CytD (2 µM) 30 min before adding the proteins for each time point. In all experiments, untreated iMDDCs were used as negative control.

### Flow cytometry

Cells were harvested after incubation with AF 488 labeled proteins and stained with LIVE/DEAD Aqua stain (Molecular probes, Thermo Fisher) or fixable viability stain 780 (BD Bioscience, San Jose, Calif., US). After this, the samples were fixed with 2% PFA and surface markers (CD11c-PE-Cy7 (clone B-ly6), CD14-APC (clone M5E2), both from BD Bioscience) were stained. At least 10′000 single cell events (according to the gating strategy, Supplementary Fig. S5) were collected on a FACSCanto II flow cytometer using the BD FACS Diva software (BD Bioscience). Data analysis was performed using FlowJo version 10 software (FlowJo LLC, Oregon, US).

### SDS-PAGE and Immunoblot analysis of α-Gal proteins

After incubation with AF 488 labeled BSA and BSA-α-Gal for 1, 2, 4, 6, 8 and 16 h, iMDDCs were pelleted and the cell pellets were treated with radioimmunoprecipitation lysis buffer. Unstimulated cells were used as control. The lysates were clarified by centrifugation and the soluble fractions were separated by SDS-PAGE under reducing conditions. Intracellular AF 488 labeled proteins were visualized on a ChemiDoc system (Bio-Rad, Hercules, CA, US). Following visualization, resolved proteins were transferred to polyvinylidene difluoride membranes (0.2 µm pore size) using a Bio-Rad turbo system. The membranes were probed with a previously validated^24^ monoclonal anti-α-Gal antibody (M86, Enzo Life Sciences, Farmingdale, NY, USA) followed by alkaline phosphatase labeled goat anti-mouse IgM antibody (Southern Biotech, Birmingham, AL, USA). Immunoreactive bands were visualized using nitro blue tetrazolium and 5-bromo-4-chloro-3-indolyl phosphate substrates (Bio-Rad).

### Confocal Laser Scanning Microscopy

iMDDCs were cultured for 1 h at 37 °C on coverslips coated with poly-L-lysine (Neuvitro Corporation, WA, USA) to allow cell attachment. After 1 and 4 h of incubation with AF 488 labeled proteins (10 µg/10^6^ cells), the coverslips were washed with PBS and stained with purified anti-human HLA-DR antibody (clone L243, BioLegend, San Diego, CA, USA), followed by a secondary goat anti-mouse IgG antibody conjugated with AF 555 (clone Poly4053, BioLegend). The coverslips were mounted using Prolong Gold Antifade reagent with DAPI (Molecular probes). Confocal Laser Scanning Microscopy (CLSM) was performed using the LSM 510 Meta system (Carl Zeiss, Jena, Germany) individually modified to allow imaging with improved detection efficiency using avalanche photodiodes^25^. The alpha Plan-Fluar 100 ×/1.45 oil immersion objective (Zeiss MicroImaging GmbH, Jena, Germany) was used throughout. Images were acquired at 1024 × 1024 pixel resolution, scanning speed 12.6 µs*/*pixel, without averaging, and prepared for publication using the Zeiss LSM Image Browser software.

### Statistical analysis

Data are presented as mean value ± s.e.m. Differences in protein uptake at the same time point were analyzed by two-way ANOVA using Bonferroni post hoc test. Differences in protein uptake at different time points were analyzed by two-way ANOVA using Tukey post hoc test. The effect of inhibition and uptake for red meat allergic patients was analyzed by paired two-tailed t-test. Differences were considered significant if P < 0.05. For statistical analysis GraphPad Prism version 6.00 software for Windows (GraphPad Software, La Jolla, CA, USA) was used.

## Electronic supplementary material

Supplementary information

## Data Availability

No datasets were generated or analyzed during the current study.
