# Supplementary material for: α-Gal on the protein surface affects uptake and degradation in immature monocyte derived dendritic cells
Source: Sci Rep. 2018 Aug 23;8:12684. doi: 10.1038/s41598-018-30887-8 (PMC6107510; doi:10.1038/s41598-018-30887-8)
Supplement: Supplementary file 1 — Supplementary information [file 41598_2018_30887_MOESM1_ESM.pdf]

Supplementary information

α-Gal on the protein surface affects uptake and degradation in immature monocyte derived dendritic cells

M. Krstić Ristivojević[1,2,†], J. Grundström[1,†], TAT. Tran[1], D. Apostolović[1], V. Radoi[3], M. Starkhammar[4] V. Vukojević[3], T. Ćirković Veličković[2,5], C. Hamsten[1], M. van Hage[1,*]

[1]Department of Medicine Solna, Immunology and Allergy Unit, Karolinska Institutet, and University Hospital, Stockholm, Sweden,
[2]Center of Excellence in Molecular Food Sciences, Faculty of Chemistry, University of Belgrade, Belgrade, Serbia,
[3]Department of Clinical Neuroscience, Center for Molecular Medicine (CMM), Karolinska Institutet, Stockholm, Sweden.
[4] Department of Internal Medicine, Södersjukhuset, Stockholm, Sweden
[5]Ghent University Global Campus, Yeonsu-gu, Incheon, South Korea,
† Shared first authorship
* Corresponding author

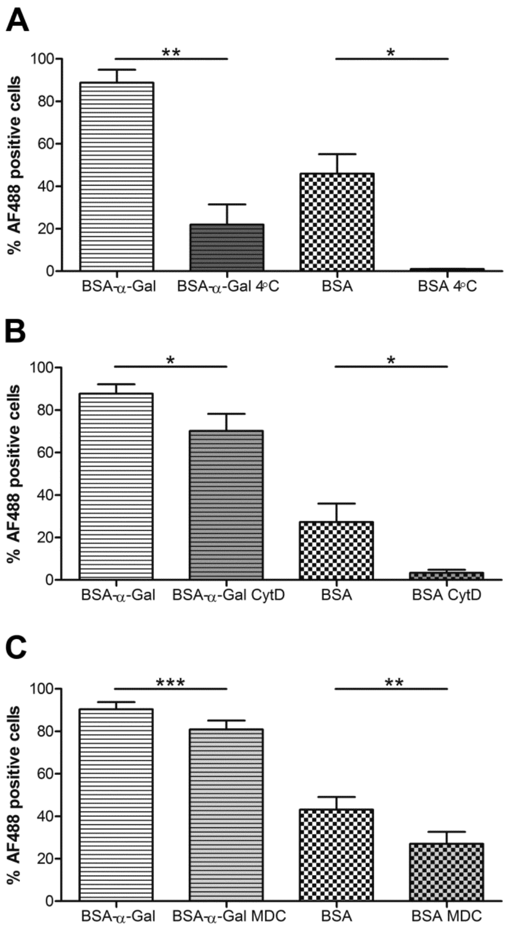

Supplementary figure S1. Inhibition of internalization of BSA-α-Gal and BSA in healthy iMDDCs after 4 h of incubation A) at 4°C, n = 4, B) with Cytochalasin D, n = 5 (BSA) and n = 6 (BSA-α-Gal), and C) with Monodansylcadaverine, n = 7.* = $p < 0.05$, ** = $p < 0.01$ and *** = $p < 0.001$ analyzed by paired t-test.

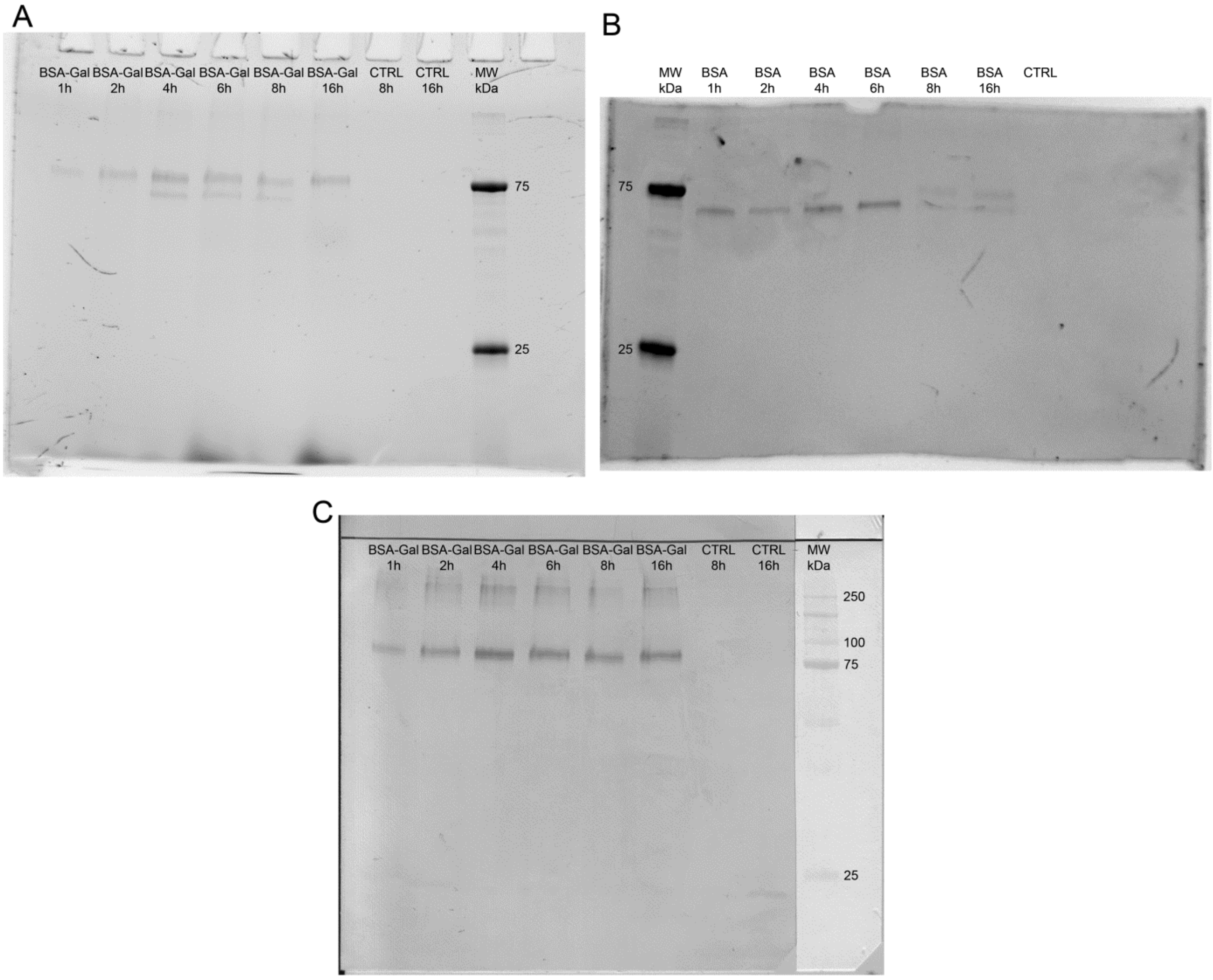

Supplementary Fig. S2. A) Fluorescent detection of BSA-α-Gal and BSA in iMDDCs lysates resolved on an SDS-polyacrylamide gel. B) Western blot detection of the α-Gal epitope in lysates from iMDDCs. iMDDCs were generated from healthy blood donors.

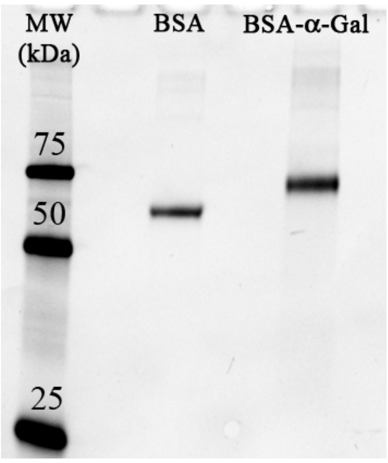

Supplementary figure S3. BSA and BSA-α-Gal resolved on a 12% polyacrylamide gel after SDS-PAGE.

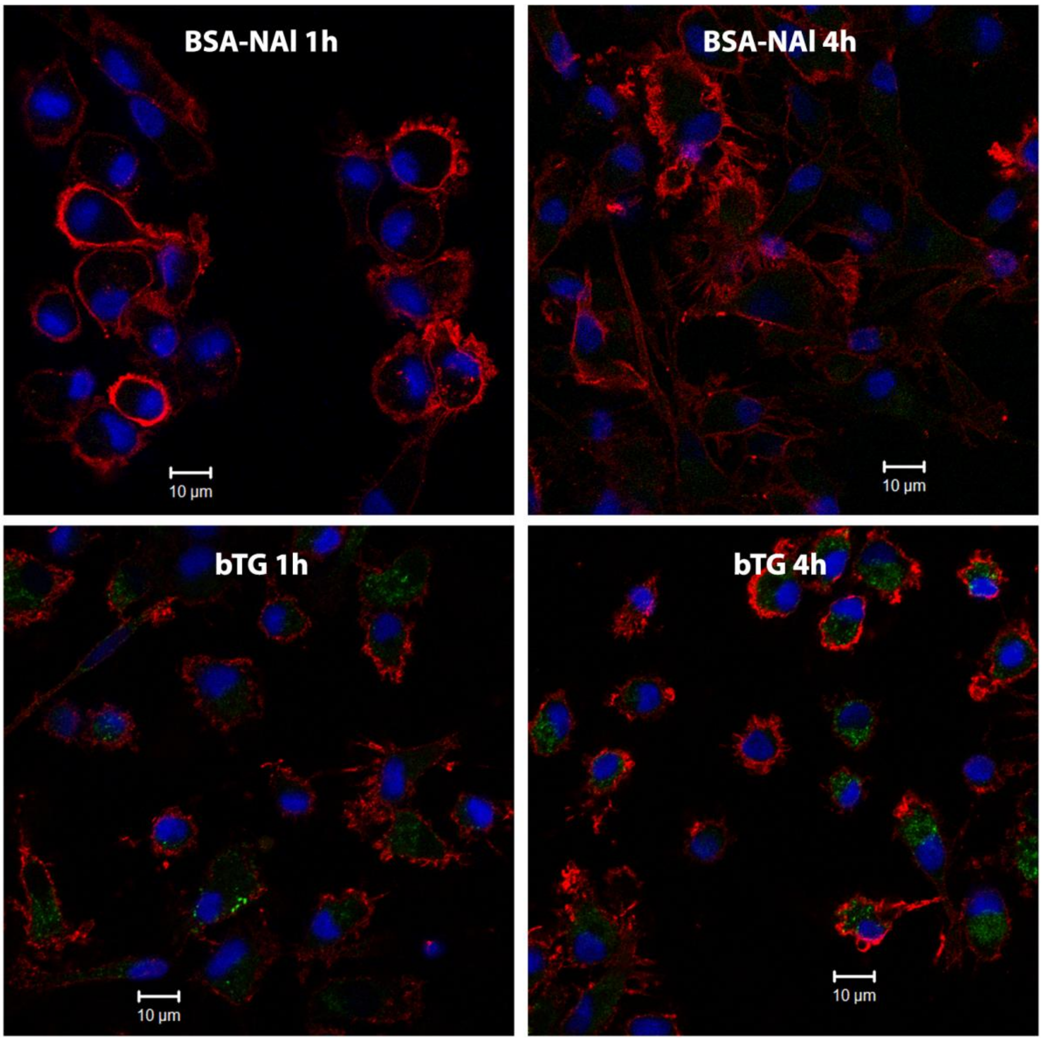

Supplementary figure S4. Uptake of BSA-Nal (top) and bTG (bottom) after 1 h (left) and 4 h (right) of iMDDC incubation at 37°C analyzed by confocal laser scanning microscopy. Green = BSA-NAl or bTG, red = HLA-DR and blue = DAPI stained nuclei.

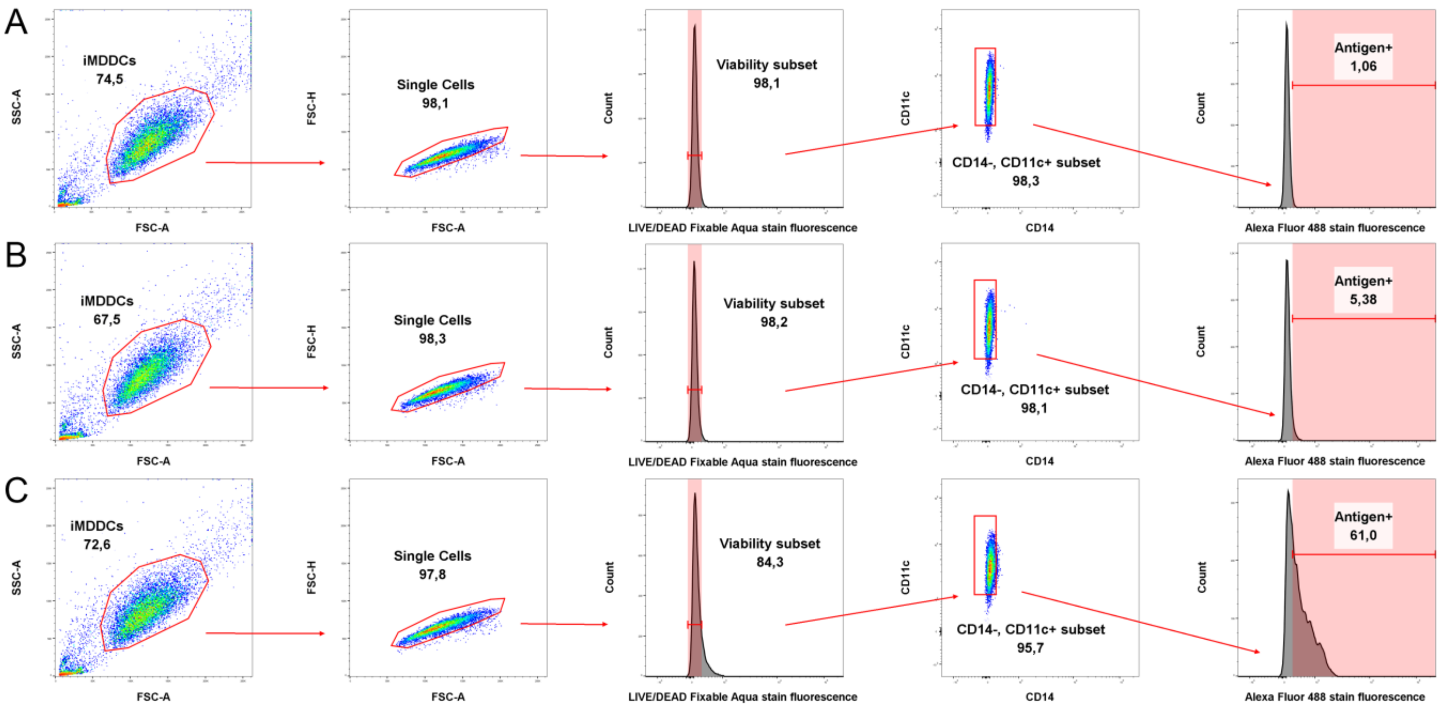

Supplementary figure S5. Gating strategy for assessment of protein uptake by iMDDCs. A) Negative control, B) BSA and C) BSA-α-Gal. Data analysis was performed using FlowJo version 10 software.
